# Pyrazinamide may possess cardioprotective properties

**DOI:** 10.1038/s41429-019-0202-z

**Published:** 2019-06-27

**Authors:** Sharabh Sinha, Qingyou Du, Sofija Jovanović, Andriy Sukhodub, Aleksandar Jovanović

**Affiliations:** 1 Division of Molecular and Clinical Medicine, Medical School, University of Dundee, Nethergate, Dundee, DD1 4HN UK; 2 University of Nicosia Medical School, Egkomi, Cyprus; 3 Center for Neuroscience and Integrative Brain Research (CENIBRE), University of Nicosia Medical School, Egkomi, Cyprus

**Keywords:** Drug regulation, Preclinical research

## Abstract

Pyrazinamide is an anti-tubercular agent, used as a part of a three-drug regime (any three of the following: rifampicin, isoniazid, pyrazinamide, streptomycin or ethambutol) for the initial phase of treatment. One of the effects pyrazinamide has on mammalian cells is to regulate NAD^+^/NADH levels. We have recently found that changes in NAD^+^/NADH are associated with regulation of expression levels of SUR2A, a cardioprotective protein serving as a regulatory subunit of cardiac ATP-sensitive K^+^ (K_ATP_) channels. Here, we have tested whether pyrazinamide regulate expression of SUR2A/K_ATP_ channel subunits and resistance to metabolic stress in embryonic heart-derived H9c2 cells. We have found that 24-h-long treatment with pyrazinamide (3 mcg/ml) increased mRNA levels of SUR2A, SUR2B and Kir6.1 without affecting mRNA levels of other K_ATP_ channel subunits. This treatment with pyrazinamide (3 mcg/ml) protected H9c2 cells against stress induced by 10 mM 2,4-dinitrophenol (DNP). The survival rate of DNP-treated cells was 45.6 ± 2.3% (*n* = 5) if not treated with pyrazinamide and 90.8 ± 2.3% (*n* = 5; *P* < 0.001) if treated with pyrazinamide. We conclude that pyrazinamide increases resistance to metabolic stress in heart H9c2 cells probably by increasing SUR2A and SUR2B expression. Our results of this study indicate that pyrazinamide should be seriously considered as a drug of choice for patients with tuberculosis and ischaemic heart disease.

Pyrazinamide is used as a part of a three-drug regime (three of rifampicin, isoniazid, pyrazinamide, streptomycin or ethambutol) for the initial phase of treatment of tuberculosis. It is unclear how pyrazinamide or its active form, pyrazinoic acid, induces their bactericidal effect on tubercle bacilli. Two separate theories have been proposed, the first suggests that pyrazinamide inhibits fatty acid synthase 1 activity to prevent the synthesis of mycolic acid while the second explains that pyrazinamide reduces the membrane potential of the bacterial cell wall and prevents transport of nutrients into *Mycobacterium tuberculosis* for RNA and protein synthesis. In mammalian cells, pyrazinamide is known to regulate intracellular NAD and NADH levels. The drug can either increase the NAD^+^ level possibly by inhibiting α-amino-β-carboxymuconate-ε-semialdehyde dehydrogenase activity (an enzyme involved in the conversion of tryptophan to niacin) or decrease the level by potentially blocking the enzyme required for the biosynthesis of NAD from niacin, nicotinic acid phosphoribosyltransferase [1].

We have shown that increased NAD + levels and NAD/NADH ratio increase intracellular levels of SUR2A, an ABC protein serving as a regulatory subunit of sarcolemmal ATP-sensitive K^+^ (K_ATP_) channels [2–4]. An increase in intracellular SUR2A level increases levels of fully assembled K_ATP_ channels, which, in turn, confers cardioprotection [5]. On the other hand, a decrease in SUR2A levels results in increased cardiac susceptibility to metabolic stress [6]. As pyrazinamide affects NAD and NADH levels, it could affect the level of SUR2A as well. If it does, it could be cardioprotective. If it is cardioprotective by regulating SUR2A, it could be seriously considered as a candidate drug against heart ischaemia. This is particularly attractive proposition as upregulation of SUR2A is more and more viewed as a promising therapeutic strategy against heart ischaemia. Minimally, if pyrazinamide is cardioprotective, it could become a drug of choice for TBC patients with cardiac ischaemia.

Experiments were performed on rat embryonic heart H9c2 cells (ECACC, Salisbury, UK) cultured at 5% CO_2_ containing Dulbecco’s modified Eagle’s medium supplemented with 10% fetal calf serum (FCS) and 2 mM glutamine. For the experiments H9C2 cells were either treated with pyrazinamide (3 μg ml^−1^) and/or vehicle. This particular concentration of pyrazinamide was selected based on our preliminary study demonstrating that this is the concentration that provides the maximal effect of cytoprotection. Also, 3 μg ml^−1^ of pyrazinamide is readily achieved in the blood when tuberculosis is treated with this drug [7]. As we have previously shown that mRNA levels of K_ATP_ channel subunits correspond to their protein levels, we have assessed mRNA levels by real time RT-PCR as we described earlier [8–10]. Briefly, for each of the primers, an RT-PCR standard curve (used to test primer efficiency) and a melting curve (used to test primer specificity) were obtained. All melting curves for the primers showed a single peak and the RT-PCR efficiency of the primers were: 100.1% for SUR1, 86.1% for SUR2A, 98.0% for SUR2B, 96.5% for Kir6.1, 100.8% for Kir6.2 and 94.5% for creatine kinase (CK). The relative expression ratio (R) of genes was calculated using 2^−ΔΔCt^ method [11].

The survival of H9C2 cells were assayed using the Multitox-Fluor Multiplex Cytotoxicity Assay (Promega). Briefly, H9C2 cells were plated in complete media (DMEM containing 10% FCS) in 96-well plates, pyrazinamide (3 μg ml^−1^) or vehicle (controls) was added to the wells. To induce severe metabolic stress, 2,4-dinitophenol (DNP), an inhibitor of oxidative phosphorylation was added to each well at the final concentration of 10 mM 24 h later. Cell survival was measured 6 h later using the peptide substrate (GF-AFC) that can be cleaved only by live cells. Following 30-min-long incubation at 37 °C, plates were measured using 1420 multilabel counter (Victor) plate reader, with excitation at 370 nm and emissions of 480 nm. The percentage of live cells was calculated based on the intensity of fluorescence according to the manufacturer's instructions [10]. All data are presented as mean ± S.E.M, with *n* representing the number of independent experiments. Mean values were compared by the ANOVA followed by Student’s *t*-test or by Student’s *t*-test alone where appropriate using SigmaStat program (Jandel Scientific, Chicago, IL). *P* < 0.05 was considered statistically significant.

In previous studies it has been shown that NAD/NADH ratio might regulate the expression of SUR2A and K_ATP_ channels [2–4]. Therefore, we have measured mRNA levels of K_ATP_ channel subunits in untreated and pyrazinamide (3 μg ml^−1^) pretreated H9c2 cells using real time RT-PCR. This method revealed that pre-treatment with pyrazinamide has significantly increased mRNA levels of SUR2A, SUR2B and Kir6.1 (cycling thresholds: SUR2A: 34.07 ± 0.10 in control cells and 33.58 ± 0.14 in pretreated cells, *n* = 6 for each, *P* = 0.04; SUR2B: 27.60 ± 0.12 in control cells and 26.97 ± 0.11 in pretreated cells, *n* = 6 for each, *P* < 0.01; Kir6.1: 25.78 ± 0.07 in control cells and 25.13 ± 0.06 in pretreated cells, *n* = 6 for each, *P* < 0.01; Fig. 1). An increase in mRNA levels was calculated to be 41.7 ± 10.6% (*n* = 6) for SUR2A, 55.2 ± 2.3% (*n* = 6) for SUR2B and 57.3 ± 4.7% (*n* = 6) for Kir6.1 (Fig. 1).

**Fig. 1:**
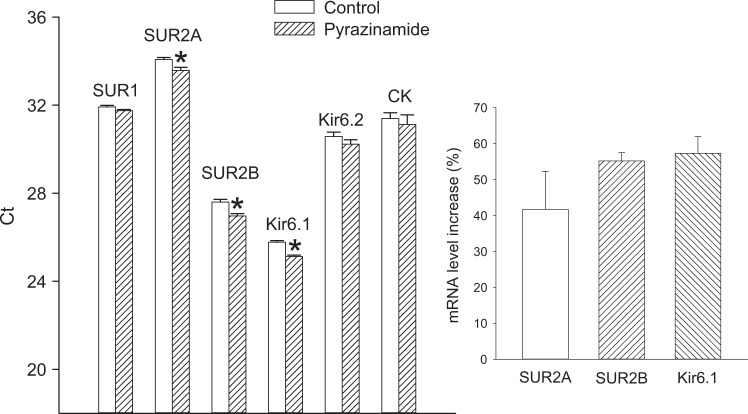
Pyrazinamide upregulates SUR2A, SUR2B and Kir6.1 in H9c2 cells. Bar graph represents cycling thresholds (Ct) of the real time RT-PCR progress curves of K_ATP_ channel subunits and creatine kinase (CK) as labelled in control cells and cells cultured with pyrazinamide (3 μg ml^−1^). Each bar represent mean ± SEM (*n* = 6 for each). *P < 0.05 when compared to control. Smaller graph represent an increase in mRNA levels calculated for K_ATP_ channel subunits with significantly different Ct values. Each bar represent mean ± SEM (*n* = 6 for each)

No statistically significant difference was observed in mRNA levels of SUR1 and Kir6.2 as well as CK (cycling thresholds: SUR1: 31.92 ± 0.07 in control cells and 31.75 ± 0.05 in pretreated cells, *n* = 6 for each, *P* = 0.34; Kir6.2: 30.58 ± 0.20 in control cells and 30.23 ± 0.20 in pretreated cells, *n* = 6 for each, *P* = 0.54; CK: 31.40 ± 0.26 in control cells and 31.13 ± 0.43 in pretreated cells, *n* = 6 for each, *P* = 0.53; Fig. 1) (Fig. 1).

DNP is a known metabolic inhibitor that is used to induce metabolic stress in different cell types. When control cells were treated with DNP (10 mM), only 45.6 ± 2.3% (*n* = 5) of cells have survived this insult (Fig. 2). Cells pretreated with pyrenzamide (3 μg ml^−1^) were significantly more resistant to DNP (10 mM) than control cells (90.8 ± 1.5% of cells pretreated with pyrazinamide have survived DNP, *n* = 5, *P* < 0.001 when compared to the control, Fig. 2).

**Fig. 2:**
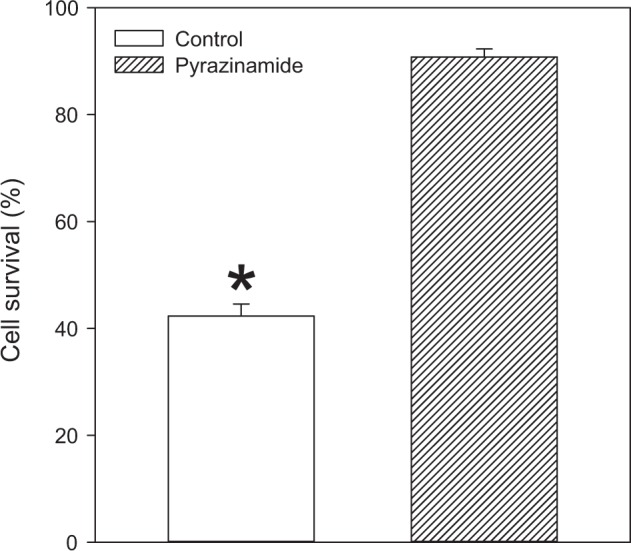
Pyrazinamide protects H9c2 cells against DNP. Bar graph showing a percentage of survival in cells cultured without and with 3 μg ml^−1^ pyrazinamide exposed to DNP (10 mM). Each bar represent mean ± SEM (*n* = 5 for each)

Pyrazinamide is a drug standardly used to treat tuberculosis as a part of a three-drug regime. It has been previously shown that in mammalian cells this antituberculotic affects levels of NAD/NADH. It has been previously demonstrated that changes in ratio of NAD/NADH regulates the level of SUR2A and, consequently, cardiac resistance to different types of metabolic stresses, including ischaemia-reperfusion [2–4]. Here, we have assessed whether pyrazinamide, that reportedly regulates NAD/NADH ratio [12, 13], would affect the level of SUR2A. We have found that this drug specifically increases SUR2A, SUR2B and Kir6.1 without affecting other K_ATP_ channel subunits. Previously it has been shown that increase in NAD^+^ concentration in vitro increases SUR2A expression which is sufficient to increase the number of fully assembled K_ATP_ channel subunits [2–4]. It has been suggested that the level of SUR2A is the rate-limiting step in formation of fully assembled K_ATP_ channels as it seems that this subunit is the least expressed of all K_ATP_ channel subunits [8]. In the present study, SUR2A expression was also increased, but, as opposed to previous studies, SUR2B and Kir6.1 were also upregulated. Whether this increase in mRNA levels of three channel subunits would result in increased resistance to metabolic stress was not clear at that point. SUR2A binds to inward rectifiers Kir6.2 and Kir6.1 to form cardiac sarcolemmal K_ATP_ channels [14]. Increased level of SUR2A in the heart protects myocardium against ischaemia-reperfusion and protects cardiomyocytes against different types of metabolic stresses [15–17]. However, it was not clear what effect on cellular resistance to stress would result when SUR2A is not increased individually, but also in combination with increase in Kir6.1 and SUR2B. It has been previously shown that concomitant overexpression of SUR2A/Kir6.2 offers less cytoprotection than overexpression of SUR2A alone [9]. On the other hand, an increase in SUR2B levels in H9c2 cells has been demonstrated to be cytoprotective [18]. DNP is a known metabolic inhibitor that was used in many studies to induce metabolic stress in different cell types [17, 18]. When applied, this compound inhibits oxidative phosphorylation resulting in decreased ATP production inducing severe metabolic stress. It has been previously shown that increased SUR2A and SUR2B each protect H9c2 cells against DNP-induced metabolic stress [9, 17]. We did not measure protein levels of SUR2A and other channel subunits ion this occasion as all studies done so far have demonstrated that changes in mRNA levels are followed up by corresponding changes in protein levels [2–4, 8, 10]. In addition, it has been shown that therapeutic strategies protecting against DNP-induced chemical hypoxia, without any exceptions reported so far, are also successful in protecting myocardium against ischaemia-reperfusion [2–4, 8, 10, 19]. It should be pointed out that the level of protection of H9c2 cells from DNP-induced stress is much higher than the level of overexpression of SUR2A alone [10]. It is possible that concomitant upregulation of SUR2B or even Kir6.1 contribute to cytoprotection afforded by pyrazinamide.

This is the first report ever to suggest that pyrazinamide is cardioprotective. Monocyte/macrophages, lymphocytes and cytokines involved in cellular-mediated immune responses against *M. tuberculosis* are also main drivers of atherogenesis [20]. Tuberculous granuloma formation affecting the coronary arteries has been described as a cause of myocardial infarction in some patients [21] and an increased risk of acute myocardial infarction in patients with tuberculosis has been reported [22]. Taken all together, cardioprotective properties of pyrazinamide should be considered when evaluating place of this drug in therapy of tuberculosis. Pyrazinamide would be obvious choice for treating patients with tuberculosis and ischaemic heart disease.
